# Thermographic follow-up of postherpetic neuralgia (PHN) subsequent to Ramsay Hunt syndrome with multicranial nerve (V, VII, VIII and IX) involvement: a case report

**DOI:** 10.1186/s12883-021-02071-5

**Published:** 2021-01-28

**Authors:** Yuan-Mei Liao, Hai-Feng Lu, Peng Xie, Ying Zhao, Qiu Han, Qian-Xi Zhang, Xiao-Hua Zuo, Yan-Na Si, Hong-Guang Bao

**Affiliations:** 1 Department of Medical Technology, Gannan Healthcare Vocational College, Ganzhou, 341000 China; 2 Department of Radiology, The Affiliated Huai’an Hospital of Xuzhou Medical University and The Second People’s Hospital of Huai’an, 223002 Huai’an, China; 3 Department of Neurosurgery, The Affiliated Huai’an Hospital of Xuzhou Medical University and The Second People’s Hospital of Huai’an, Huai’an, 223002 China; 4 Department of Neurology, The Affiliated Huai’an Hospital of Xuzhou Medical University and The Second People’s Hospital of Huai’an, Huai’an, 223002 China; 5 Department of Neurology, Huai’an First People’s Hospital, The Affiliated Huai’an NO.1 People’s Hospital of Nanjing Medical Universtiy, Huai’an, 223002 China; 6 Department of Anesthesiology, Nanjing First Hospital, Nanjing Medical University, Nanjing, 210006 China; 7 Department of Pain Management, The Affiliated Huai’an Hospital of Xuzhou Medical University and The Second People’s Hospital of Huai’an, Huai’an, 223002 China

**Keywords:** Postherpetic neuralgia (PHN), Ramsay Hunt syndrome, Multiple cranial nerves, Linear-polarized near-infrared light, Infrared thermography

## Abstract

**Background:**

Ramsay Hunt syndrome (RHS) is caused by a reactivation of varicella-zoster virus (VZV) infection, and it is characterized by the symptoms of facial paralysis, otalgia, auricular rash, and/or an oral lesion. Elderly patients or immunocompromised patients, deep pain at the initial visit and no prompt treatment are significant predictors of postherpetic neuralgia (PHN). When PHN occurs, especially involved cranial polyneuropathy, multiple modalities should be administered for patients with the intractable PHN. The use of thermography in the follow-up of PHN secondary to RHS with multicranial nerve involvement has not yet been described yet in the literature.

**Case presentation:**

The patient was a 78-year-old man with the chief complaint of a 3-month history of PHN secondary to RHS with polycranial nerve (V, VII, VIII, and IX) involvement. Multimodality therapy with oral gabapentin, pulsed radiofrequency (PRF) application to the Gasserian ganglion for pain in the trigeminal nerve region, linear-polarized near-infrared light irradiation for pain in the facial nerve region, and 2% lidocaine spray for pain in the glossopharyngeal nerve region was used to the treat patient, and follow-up evaluations included thermography. This comprehensive treatment obviously improved the quality of life, resulting in considerable pain relief, as indicated by a decrease in the numerical rating scale (NRS) score from 9 to 3 and a decrease in thermal imaging temperature from higher to average temperature on the ipsilateral side compared with the contralateral side. Lidocaine spray on the tonsillar branches of the glossopharyngeal nerve resulted in an improvement in odynophagia, and the NRS score decreased from 9 to 0 for glossopharyngeal neuralgia after three applications.

**Conclusion:**

Although the use of thermography in the follow-up of RHS with multiple cranial nerve (V, VII, VIII, and IX) involvement is very rare, in this patient, thermal imaging showed the efficacy of combination therapy (oral gabapentin, 2% lidocaine sprayed, PRF application and linear-polarized near-infrared light irradiation) and that is a good option for treatment.

## Background

Ramsay Hunt syndrome (RHS) is a multicranial neuropathy caused by the varicella-zoster virus (VZV) infection, and it is characterized by the symptoms of facial paralysis, otalgia, auricular rash, and/or an oral lesion [1, 2]. Correct diagnosis and subsequent proper treatment can reduce the incidence of postherpetic neuralgia (PHN), which may lead to significant physical, occupational, and social impairments due to the unceasing pain [3]. Elderly patients or immunocompromised patients, deep pain at the initial visit or a lack of proper therapy at the beginning of the illness are significant predictors for PHN [4]. When PHN occurs, especially involved cranial polyneuropathy, multiple modalities should be administered for patients with intractable PHN.

To monitor the response to treatment in patients with PHN, infrared thermography (a noninvasive medical diagnostic tool for analysing the physiological function of the sensory nervous system, autonomic nervous system, and local and systemic inflammation) is used to study the physiology of thermoregulation and reveals a correlation of thermal imaging with the intensity of pain. Infrared thermography has been shown to quickly detect someone’s surface skin temperature without being physically close to the person being evaluated for the duration of the public health emergency related to coronavirus disease 2019 (COVID-19) [5]. Previously, studies were performed on RHS associated with multiple cranial nerves (CN IV, CN V, CN VII-XI, and cervical nerves C2 - C3) [6, 7], and thermographic follow-ups in herpes zoster and PHN [8, 9]. Nevertheless, PHN secondary to RHS associated with multicranial nerve (CN V, CN VII, CN VIII, and CN IX) involvement and follow-up with infrared thermal has not yet been described yet in the literature. In this paper, the improvement in PHN after comprehensive treatment was evaluated according to the NRS score and infrared thermography.

## Case presentation

**Fig. 1:**
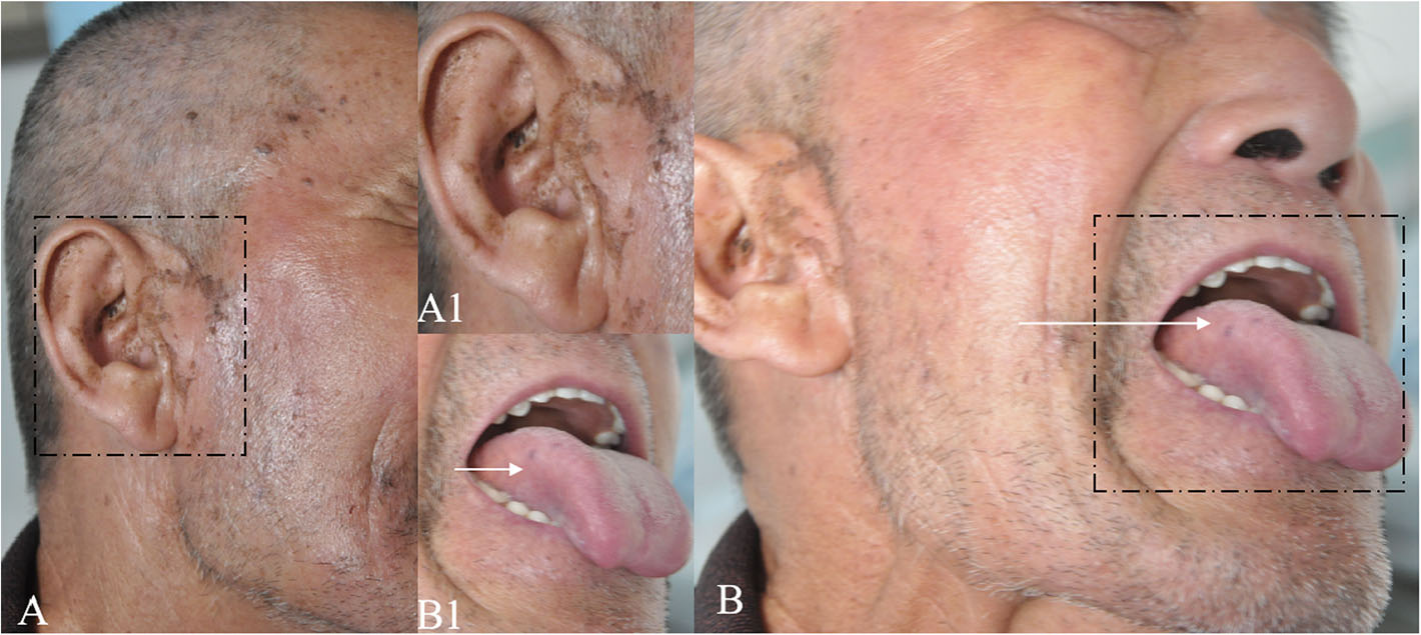
The Patient exhibited a herpes zoster scar of the right auricle (A, A1), right temporal region (A), and right side of tongue root (B, B1, arrow)

A 78-year-old man with the a chief complaint of a 3-month history of gradually increasing pain and numbness in the right ear, submandibular region, temporal region, and the right side of the tongue root and right-sided facial weakness was seen (Fig. 1). Paroxysms of odynophagia triggered by swallowing lasted throughout mealtime. Depending on swallowing and stress, the pain intensity was rated a 9–10 on the numerical rating scale (NRS). The patient had been almost unable to take any food for several days. He complained of otalgia and hot flashes on the right side of the face and ear. He had not had symptoms of vertigo, tinnitus, nausea, or vomiting. Peripheral facial paralysis was classified as House-Brackmann grade III. The results of the audiogram were normal. Laboratory investigations and brain magnetic resonance imaging (MRI) were unremarkable. Medical therapy began with the application of ipsilateral lidocaine spray to the tonsillar branch of the glossopharyngeal nerve before mealtime. The second day after three lidocaine sprayed applications, the patient’s odynophagia intensity decreased from a score of 9 to 0 rating on the NRS. The recommended dosage was adjusted according to the response, gabapentin was initiated at a dose of 300 mg once daily, and then the dosage was increased by 300 mg every day until a therapeutic dose (600 mg three times daily) was reached.

**Fig. 2:**
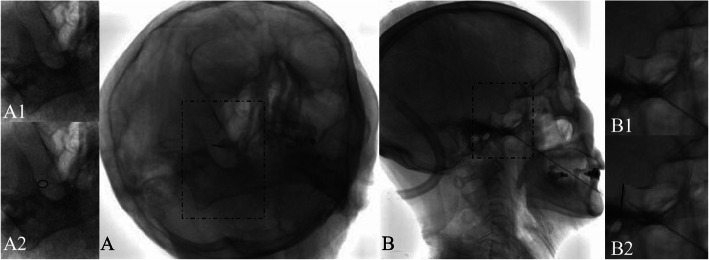
Under C-arm guidance, the needle is safely advanced until its tip is positioned in the foramen ovale anteroposterior (A, A1, A2) and equal to the lateral clivus (B) lateral

**Fig. 3:**
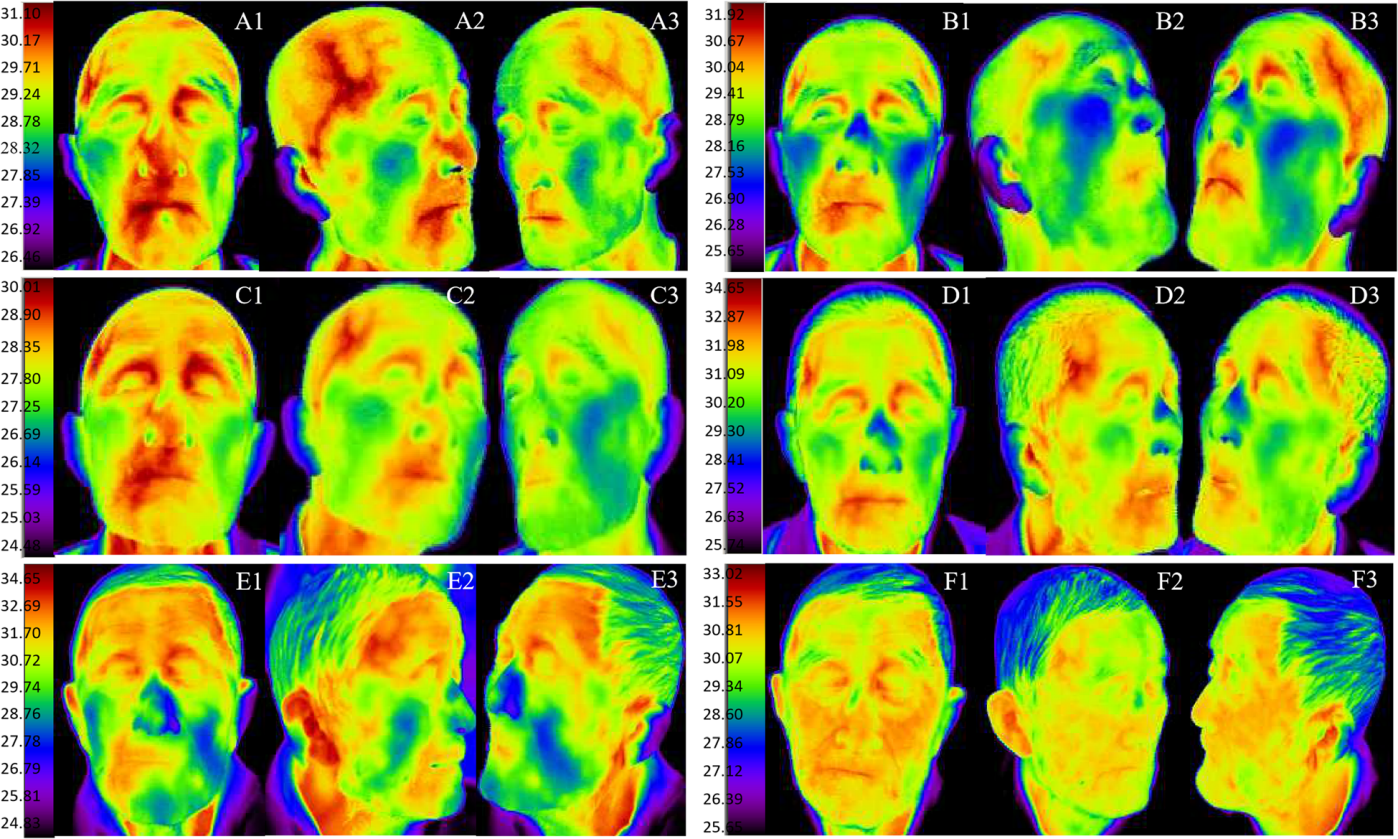
Thermographic follow-up of postherpetic neuralgia (PHN). Infrared thermograms of lesions and contralateral sides before treatment (A1, A2, A3); one week after the first pulsed radiofrequency (PRF) application (B1, B2, B3); one week after the second PRF application (C1, C2, C3). Infrared thermography showed better performance than contralateral thermography at one week, one month (D1, D2, D3), and three months. Thermographic findings of the pain area showed a higher temperature than that of the the contralateral side at three months when a dose of gabapentin was missed (E1, E2, E3), whereas thermography showed a lower temperature image after gabapentin administration (F1, F2, F3). Scale numbers adjacent to their corresponding colours represent body temperature in degrees Celsius

The delivery of pulsed radiofrequency (PRF) to the Gasserian ganglion was recommended to alleviate the pain of the mandibular nerve division of the trigeminal nerve. A radiofrequency generator (ET-20S, Smith & Nephew, Massachusetts, USA) was inserted through the foramen ovale into Meckel’s cave under common fluoroscopic control guided by bony landmarks (Fig. 2). Sensory testing at 50 Hz and motor testing at 2 Hz were performed to determine whether the needle was on the sensory target. The PRF was thermally coagulated at 42 °C for 120 s, at two bursts per second of 20 milliseconds duration. The first PRF application to the Gasserian ganglion produced some pain relief; then, the second PRF application to the Gasserian ganglion was performed a week later. Pain intensity was correlated with thermal imaging. Infrared thermography of the treated pain area did not reveal a higher temperature than that of the contralateral side at one week, one month, or three months (Fig. 3b; c; d; f); Thermographic findings of the pain area showed a higher temperature than that of the contralateral side at three months when a dose of gabapentin was missed (Fig. 3e), whereas thermography showed a lower temperature image after gabapentin administration.

Peripheral facial paralysis was treated with linear-polarized near-infrared light irradiation [10] (MINATO ALB-200H, Minato, Japan) under the following conditions: a power of 1.4 W, a cycle of 1 s on and 3 s off; and a duration of 10 min, once a day, and ten times in all. The House-Brackmann grade at one month indicated that the patient was healthy. The lesions were followed up by thermogram (IRIS-5000, Medicore, Seoul, South Korea) during combined therapy and after one week, one month and three months.

Written informed consent was obtained from the patient for the publication of this case report and accompanying images.

## Discussion and conclusion

RHS with simultaneous involvement of multiple cranial nerve ganglia (the geniculate ganglion and peripheral ganglia of cranial nerves VIII, IX, and X) caused by VZV infection and its subsequent activation may cause herpes zoster cephalicus, facial paralysis, otalgia, an auricular rash and/or an oral lesion [11]. In 1915, Sharpe classified herpes zoster cephalicus into five categories based on the inflammation of the geniculate, auditory, glossopharyngeal or vagal ganglia with or without concomitant facial and acoustic symptoms [11]. Hunt’s original description in 1907 included only the inflammation of sensory ganglia of the cranial nerves CNVII, CN VIII, CN IX, and CN X. The original description did not adequately address the polyneuropathic pathogenesis or outlined classification of this syndrome, for example, central (CN V, CN IX, CN X, CN XI, and CN XII), cervical (spinal nerves C2 and C3) and peripheral nerves were not included in the original description and nor was a description of the co-involvement cranial nerves. Although the mechanism of this involvement remains unclear, there are three common hypotheses to explain this relationship. First, in the embryological and anatomical correlation hypothesis, the vestibulocochlear nerve is located near the Geniculate ganglion within the bony facial canal; therefore, inflammation in a single ganglion could spread in a chain-like manner to adjacent ganglia, including the Gasserian, geniculate and other cranial and second/third cervical ganglia. Second, in the vasculitis hypothesis, contiguous cranial neuropathies may be partly due to the selective vulnerability of blood vessels to varicella-zoster virus and the universal blood supply. Finally, in the brainstem reflex pathways hypothesis, VZV is hypothesized to spread along brainstem reflex pathways through an intersynaptic transmission, mainly in an anterograde direction, resulting in cranial polyneuropathy [12].

When PHN occurs, resulting in severe physical, occupational, and social impairments due to unceasing pain, combination therapy should be administered to patients with intractable PHN. First, the application of lidocaine spray to the tonsillar branches of the glossopharyngeal nerve resulted in complete resolution of the lateral glossopharyngeal neuralgia due to Eagle’s syndrome [13]. Topical therapeutic approaches produce lower systemic concentrations, which result in fewer systemic side effects and drug-drug interactions. Lidocaine is a local anaesthetic that modulates pain by blocking the transmission of impulses in peripheral nerves through the blockade of voltage-gated sodium channels [14]. Topical anaesthetics can also suppress the phosphorylation of extracellular signal-regulated kinases (ERKs) in the dorsal horn of the spinal column, which plays a critical role in signal transduction of the pain pathway [15, 16]. The use of topical anaesthetics could relieve the pain of neuropathic pain or pain of PHN through a combination of central and peripheral nervous system actions [17]. Treatments for postherpetic (glossopharyngeal) neuralgia include the application of local lidocaine sprays rather than the invasive treatment, which can lead to arterial hypotension, neuritis, deafferentation pain, neurovascular injury, and severe haemodynamic problems [18]. Noninvasive local anaesthetics sprays should be considered as one of the alternative treatments (more than a diagnostic test for the glossopharyngeal neuralgia) for glossopharyngeal neuralgia caused by herpes zoster. Our results showed that lidocaine spray application on the tonsillar branches of the glossopharyngeal nerve resulted in the relief from odynophagia, and the NRS score decreased from 9 to 0 for the glossopharyngeal neuralgia after three applications. A glossopharyngeal neuralgia patient relieved pain by using topical lidocaine sprayed before mealtime for six years. Second, linear-polarized near-infrared light irradiation has both light chemical and thermal effects on the neuromuscular system and alleviates pain, and it has some advantages, such as its noninvasiveness, easy and straightforward operation, and shorter time per treatment session than electrogalvanic stimulation [19]. This therapeutic modality, sometimes substituted for invasive treatment in patients, has been used effectively to treat painful disorders, such as temporomandibular joint pain, frozen shoulder, osteoarthritis, rheumatoid arthritis, thalamic pain, complex regional pain, intractable anorectal pain, and PHN [20]. Linear-polarized near-infrared light irradiation is an important treatment modality for patients recovering from Bell’s palsy (neuronal recovery and maintaining facial muscle tone), and it stimulates the peripheral nervous system resulting in vasodilation, muscle relaxation and the normalization of the autonomic nervous system [10, 21]. Our results showed that the House-Brackmann score changed to Grade I at one month following linear-polarized near-infrared light irradiation.

Infrared thermography was used to study the physiology of thermoregulation and revealed a correlation of thermal images with the intensity of pain [9]. Warm spots observed via infrared thermography reflect inflammation as a result of histamine or substance P secretion, whereas cold spots reflect sympathetic nerve activity and sweating. When combination therapy is prescribed, the right-sided infrared thermographic image of the pain region did not show a higher temperature than the thermographic image of the contralateral side at one week, one month, and three months; therefore, thermography might be a useful tool to objectively assessment tool of subjective pain symptoms in patients with PHN. The first PRF application to the Gasserian ganglion produced some pain relief; then, the second PRF application to the Gasserian ganglion was performed a week later. Pain intensity was correlated with thermal images. The infrared thermogram of the treated area did not reveal a higher temperature than that of the contralateral side at one week, one month, and three months. Especially when a dose of gabapentin was missed, thermographic findings showed a higher temperature in the pain zone than in the contralateral side at three months. Patient treatment can be adjusted according to infrared thermographic findings and the NRS score. Gabapentin should not be stopped abruptly; it should be reduced gradually over a minimum of one week [22].

This comprehensive treatment improved the quality of life, provided considerable pain relief, and showed a correlation of thermal images with the intensity of pain. Further prospective investigations might be needed to estimate the effectiveness of comprehensive treatment for polycranial nerve PHN secondary to RHS with multicranial nerve (CN V, CN VII, CN VIII, and CN IX) involvement.

## Data Availability

The datasets used and/or analysed during the current study are available from the corresponding author upon reasonable request.

## Abbreviations

PHN: Postherpetic neuralgia
RHS: Ramsay Hunt syndrome
VZV: Varicella-zoster virus
PRF: Pulsed radiofrequency
NRS: Numerical rating scale
CN: Cranial nerve
MRI: Magnetic resonance imaging
